# Analysis of the Way and Correctness of Using Automated External Defibrillators Placed in Public Space in Polish Cities—Continuation of Research

**DOI:** 10.3390/ijerph18189892

**Published:** 2021-09-20

**Authors:** Daniel Ślęzak, Marlena Robakowska, Przemysław Żuratyński, Joanna Synoweć, Katarzyna Pogorzelczyk, Kamil Krzyżanowski, Magdalena Błażek, Jarosław Woroń

**Affiliations:** 1 Department of Medical Rescue, Medical University of Gdańsk, 80-210 Gdańsk, Poland; przemyslaw.zuratynski@gumed.edu.pl (P.Ż.); kamil.krzyżanowski@gumed.edu.pl (K.K.); 2 Department of Public Health & Social Medicine, Medical University of Gdańsk, 80-210 Gdańsk, Poland; marlena.robakowska@gumed.edu.pl (M.R.); katarzyna.pogorzelczyk@gumed.edu.pl (K.P.); 3 Independent Researcher, 80-210 Gdańsk, Poland; joanna@synowec.pl; 4 Division of Quality of Life Research, Medical University of Gdańsk, 80-210 Gdańsk, Poland; magdalena.blazek@gumed.edu.pl; 5 Department of Clinical Pharmacology, Jagiellonian University, 31-531 Kraków, Poland; j.woron@uj.edu.pl

**Keywords:** automatic external defibrillator, AED in Poland, use of AEDs in Poland, correctness of AED use in Poland, first aid, AED

## Abstract

Immediate resuscitation is required for any sudden cardiac arrest. To improve the survival of the patient, a device to be operated by witnesses of the event—automated external defibrillator (AED)—has been produced. The aim of this study is to analyze the way and correctness of use of automated external defibrillators placed in public spaces in Polish cities. The data analyzed (using Excel 2019 and R 3.5.3 software) are 120 cases of use of automated external defibrillators, placed in public spaces in the territory of Poland in 2008–2018. The predominant location of AED use is in public transportation facilities, and the injured party is the traveler. AED use in non-hospital settings is more common in male victims aged 50–60 years. Owners of AEDs inadequately provide information about their use. The documentation that forms the basis of the emergency medical services intervention needs to be refined. There is no mention of resuscitation performed by a witness of an event or of the use of an AED. In addition, Poland lacks the legal basis for maintaining a register of automated external defibrillators. There is a need to develop appropriate documents to determine the process of reporting by the owners of the use of AEDs in out-of-hospital conditions (OHCA).

## 1. Introduction

Cardiovascular deaths are the most common cause of all registered deaths in Europe and the world, and the most common cause of all deaths recorded by the World Health Organization (WHO) [1,2]. Economic development rates and industrialization have significantly started to affect the number of deaths due to sudden cardiac arrest (SCA). According to Olson et al. [3], sudden cardiac arrest accounts for more than half of all cardiovascular deaths and is the first sign of heart disease in 50% of these individuals.

Immediate resuscitation, a combination of chest compressions and assisted ventilation, is required for all sudden cardiac arrests. European Resuscitation Council (ERC) and International Liaison Committee on Resuscitation (ILCOR) guidelines indicate that in addition to resuscitation, defibrillation is important as soon as possible. For every minute that defibrillation is delayed, the probability of survival after SCA decreases by 10% [2,4].

Defibrillation should be performed as quickly as possible. Therefore, to improve patient survival after sudden cardiac arrest in the out-of-hospital setting, a device intended for use by a witness to sudden cardiac arrest is required. Automated external defibrillator (AED) as a small size device, with easy and intuitive operation, can be placed in a public place. To serve its purpose, the AED should be available in areas with a high likelihood of OHCA. An AED allows defibrillation many minutes before professional help arrives. According to Kwon [5], there is a strong recommendation for the use of external defibrillators in public places and it is closely related to Return of Spontaneous Circulation (ROSC).

The aim of this study is to analyze the way and correctness of use of automated external defibrillators placed in public spaces in Polish cities. To achieve this, the following research questions were posed: How can the person in whom the use of AED was applied be characterized (age, gender, profile of the victim)? What is the profile of the place where AED was used most often? By whom was the AED device used most often? How long did it take to apply the adhesive AED electrodes to the exposed chest of the casualty? How often did the use of the AED involve performing a defibrillation? How much time elapsed from the moment the AED was turned on until the arrival of the Emergency Medical Service? Does use of the AED result in return of spontaneous circulation (ROSC) at the scene? Do the emergency notification centers and the emergency medical service (EMS) records contain information about the use of the AED?

## 2. Materials and Methods

The study material consists of data on cases of use of an automated external defibrillator in adults (over 18 years of age) in the period from 1 January 2008 to 31 December 2018 in Poland. Only cases of use of AEDs in a public place, other than a medical facility, were analyzed, along with the exclusion of emergency services, i.e., the State Fire Service and Volunteer Fire Service, which have AEDs as part of their emergency equipment.

The study was approved by the Independent Bioethics Committee for Scientific Research at the Medical University of Gdansk No. NKBBN/52/2018 dated 12 February 2018. The data analyzed represent 120 cases of use of an automated external defibrillator placed in public spaces between 2008 and 2018. In addition, the research material were responses from a diagnostic survey questionnaire. The author’s questionnaire was sent to units where AEDs are located. The respondents’ answers to the questions recorded in the questionnaire were consistent with their consent to participate in the voluntary and free survey. The questionnaires were sent electronically. The questionnaire included the following questions:

1. Have any of the AED devices placed been used (years 2008–2018)?
2. By whom was the AED device used?
3. Under what circumstances was the AED device used? Please briefly characterize.
4. Was the AED distributor/manufacturer informed of the device’s use?
5. Did the AED distributor/manufacturer assist you in any way?

The next sources of data were AED manufacturers and distributors in the country, viz: Cardiac Science, Max Harter, Medline, Paramedica Polska, AEDMAX, Stryker, HS Medical, Medtronic, PHYSIO-CONTROL POLAND, Philips, DefiMed, Emtel, HeartSine, DefibTech, IPad, Primedic, Anatom. The respondents’ answers to the questions written in the questionnaire were consistent with their consent to participate in the voluntary and free study. The questionnaires were sent electronically. In case of no response, another e-mail was sent after one month. The questionnaire sent to manufacturers and distributors of AEDs in Poland contained the following questions:

1. Do you have knowledge about the use of AED device (years 2008–2018)?
2. Under what circumstances has the AED device been used? Please briefly characterize.
3. Did the device manager inform you of the use?
4. Did the AED manager require any assistance from you?

Another source of data analyzed were the responses from the author’s questionnaire sent to foundations involved in promoting first aid and healthy living principles such as: Great Orchestra of Christmas Charity Foundation, Polish Red Cross, World to Children Foundation, ORLEN “Gift of the Heart” Foundation, Department of Bioinformatics and Telemedicine of Collegium Medium UJ in Krakow. The questionnaire contained the following questions:

1. Do you have knowledge about the use of AED device (2008–2018)?
2. Under what circumstances has the AED device been used? Please briefly characterize.

A retrospective analysis of selected medical records was also conducted. Data were obtained from the reports of the emergency notification center (medical dispatch center), cards of medical rescue activities—after obtaining permission from the dispatcher of the state medical rescue system—and the medical dispatch center. The data obtained were anonymized using only the date and time of the event, age, gender, implemented procedure. We analyzed data generated from a specific “type” of patients, from a specific unit in a given period (giving dates, months, or years), retrospectively in relation to the years 2008–2018.

The research material was collected in Microsoft Office Excel spreadsheet for Windows systems. Statistical analyses were performed using R 3.5.3 software (R version 3.5.3 (Great Truth) of 2019). Data testing was performed according to Poissone tests with a significance level of α = 0.05.

## 3. Results

### 3.1. Analysis of the Questionnaire of Questions Sent to the Units in the Public Space

The questionnaire was sent electronically to 1165 units in the public space that were not a treatment facility or fire station. The total amount of feedback was 326 (27.98%). Based on the questionnaire, it was determined that 65 emergency interventions with an AED occurred at 60 locations between 2008 and 2018. Of these, 55 were conducted by a unit employee (84.62%), while only 10 were conducted by a bystander (15.38%). For 34 locations where an AED was used, the unit reported the fact to the distributor/manufacturer of the device (56.67%). Every unit that reported AED use received some form of assistance from the AED distributor/manufacturer. The assistance consisted of replacing the electrodes, servicing the device, and reading out the recording of the resuscitation action.

### 3.2. Analysis of the Questionnaire Sent to AED Distributors and Manufacturers

The questionnaire was sent electronically to 17 manufacturers and distributors of AED devices in Poland. The total number of responses was 14 (82.35%). Only 5 entities provided lists of AED usesin total data from 68 interventions were obtained. Nine respondents refused to provide any information, arguing that this was due to commercial law and broadly understood personal data protection. The respondents also provided information that “the owner of the AED did not want the information about its use to be publicized (despite successful defibrillation)”. Quoting the words of one entity: “The defibrillator after the sale is the property of the entities that purchased it. All data about the course of the action (date, time, duration, rhythm diagram before and after defibrillation, number of discharges and their energy, etc.) are in the memory of the device and it is the owner who has them. Only once in our more than four years of activity, it happened that the owner of the AED asked to “dump” this data. It was at the request of a physician who took over the care of a patient on the ward”.

Based on the data obtained from manufacturers and distributors, a list of 120 AED uses was compiled. Additionally, 33 records of AED use were obtained.

### 3.3. Analysis of AED Use Cases

#### 3.3.1. Patient Characteristics

Analyzing the sex of the casualty it was noticed that most of the AED interventions concerned males (87.5%). The presentation of the age of the victim is shown in. Table 1.

**Figure 1:**
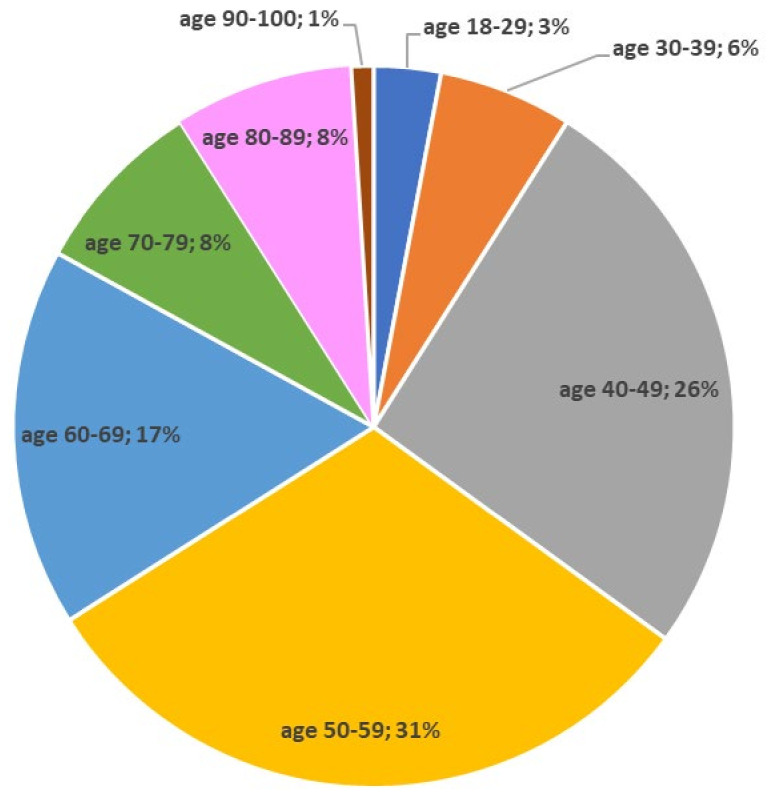
Age range of victims (n = 120).

The distribution of the data in Table 1 is symmetric with respect to the mean as the skewness value is between −2 and +2 and clustered with respect to the mean (kurtosis is between −2 and +2). For the percentage description, the age of the victims was assigned to a given age range (18–29; 30–39; 40–49; 50–59; 60–69; 70–79; 80–89; 90–100) and it was noted that the highest rate of AED use occurred in the 50–59 age group (31%) which is included in the mean (57.27) and median (56.5). The age range is presented in Figure 1.

When presenting the profile of a victim who received AED, the dominant group were travelers by different means of transport (30%). Similarly high values were noted among people classified as “client/petitioner” of a shopping, banking, or office establishment (23.33%) and “employee” (22.5%). Only 1 case was related to a sports player performing physical exercise in a sports center. Detailed results are included in Table 2.

#### 3.3.2. Characteristics of Where the AED Has Been Used

AEDs were used in the following cities: Marki, Sandomierz, Wojnicz, Mszczonów, Radom, Kraków, Warsaw, Słupsk, Bydgoszcz, Łódź, Jaworzno, Chybie, Zielona Góra, Karpacz, Wrocław, Gościeradów, Czeladź, Gdańsk, Bielsk Podlaski, Szczecin, Krosno, Katowice, Poznań, Kalisz, Grudziądz, Leszno, Orzesze, Mielec, Wolbrom, Oleśnica, Zagórów, Ryglice. The greatest number of interventions were recorded in Warsaw (17.98%) and Krakow (16.85%). In terms of provinces, the highest number of interventions with AEDs took place in Mazowieckie (21.11%), Małopolskie (20%), and Śląskie (14.44%). According to the data obtained, no AEDs were used in Opolskie.

Most interventions with use of AED occurred in the area of public transport infrastructure (24.17%). The use of AEDs was similarly frequent in factories, warehouses (workplace—17.5%). The whole cross-section of places is presented in Table 3.

#### 3.3.3. Characteristics of the Person Who Used the AED

Representing the person who used the AED during cardiopulmonary resuscitation (CPR), there is a statistically significant advantage of employees (n = 96) over casual witnesses (n = 24). Casual witnesses included paramedics, medical students, and qualified first aid rescuers, while employees were represented by security guards, police officers, medical staff, hotel staff, streetcar driver, and pool attendants.

#### 3.3.4. Analysis of Resuscitation with the Use of AED

Out of 120 cases of AED use in OHCA (out-of-hospital cardiac arrest), 76 required defibrillation (63.33%). In 37 cases (30.83%), defibrillation was not performed. Detailed information was not obtained from 7 AED interventions (5.83%). The data represent a comprehensive account of the results obtained (responses from questionnaires, bibliographic materials, electrocardiogram recordings).

On the basis of 33 thoroughly documented resuscitation actions with the use of AED it can be observed that the mean time which elapsed from SCA to the use of AED oscillated around 222.73 s (SD = 121.98 s), which converting to minutes amounts to 3 min 43 s. The median value of the parameter studied was 180 s (3 min). The maximum time that elapsed from SCA to AED use was 600 s, i.e., 10 min. Based on descriptive statistics of the studied variable, most of the results are smaller than the mean (positive skewness), i.e., below 3 min 22 s, indicating the existence of many outliers of the mean (kurtosis + 3). Table 4 presents the detailed results.

After activation of the automated external defibrillator, the self-adhesive electrodes were correctly placed on the victim’s body in an average of 35.36 s (SD = 31.17 s). The median value of the parameter studied was 22 s. The maximum elapsed time was 115 s, or 1 min 55 s. Based on the descriptive statistics of the studied variable, most of the results are smaller than the mean (positive skewness), i.e., less than 36 s, indicating the existence of many values close to the mean (positive kurtosis) (Table 4).

During the intervention, the AED performed an average of 2.79 cardiac rhythm analyses. The median was 2 analyses. For 1 intervention, 6 heart rate analyses were performed. Based on the descriptive statistics of the study variable, most of the results are smaller than the mean (positive skewness), that is, less than 2 analyses, with an indication that there are many values close to the mean (positive kurtosis) (Table 4).

For 24 interventions, the first OHCA mechanism analyzed was ventricular fibrillation (VF) which accounted for 72.73%. One case was pulseless ventricular tachycardia (VT). Non-defibrillatory rhythms were recognized for a total of 8 events asystole (AS)—n = 5, 15.15%; pulseless electrical activity (PEA)—n = 3; 9.09%). Defibrillation after the first analysis was performed for 24 events (72.73%). It is puzzling why defibrillation was not performed for VT. The time that elapsed from the end of the rhythm analysis, defibrillation recommendation to the triggering of the electrical impulse was on average 7 s (SD = 1 s). After 2 min, the AED performed another cardiac rhythm analysis (protocol compliance according to ERC 2015). In two cases there was no further recording from the AED as the casualty was handed over to the paramedics. According to the Medical Emergency Card, the identified heart rhythm from the manual defibrillator recording indicated sustained VF. Based on data from 33 recordings, the following were noted: VF—n = 16; 51.61% of cases, AS—n = 6; 19.35%, PEA—n = 1; 3.33%. In 8 interventions (25.81%) sinus rhythm was successfully restored. Defibrillation was performed in every analyzed case of VF. After another 2 min, the AED performed cardiac rhythm analysis (protocol compliance according to ERC 2015). In 9 cases there were no further recording from the AED as the casualty was handed over to the paramedics. According to the Medical Emergency Card, the identified heart rhythm based on the recording from the manual defibrillator indicated sustained VF—n = 7 or AS—n = 2. Based on the data from 14 records, the following were noted: VF—n = 6; 42.86% of cases, AS—n = 4; 28.57%, PEA—n = 1; 7.14%. Sinus rhythm was successfully restored in 3 interventions (21.43%). Defibrillation was performed in every analyzed case of VF. After another 2 min, the AED performed cardiac rhythm analysis (protocol compliance according to ERC 2015). Only 5 interventions have such long AED use times. Based on the data from the 5 records noted: VF—n = 2; AS—n = 3. The percentage presentation is not justified. In 3 interventions sinus rhythm was successfully restored. Defibrillation was performed in every VF case analyzed. Summarizing the total time of AED operation at the scene, the mean was 454.12 s (7 min 34 s) with SD = 190.62 s (3 min 10 s). The minimum time of AED operation was 131 s (1 min 11 s), the maximum—923 s (15 min 23 s). The median for the parameter studied was 478 s (7 min 58 s). Based on descriptive statistics of the tested variable, the majority of results were smaller than the mean (positive skewness), i.e., below 7 min 34 s, indicating the existence of many values close to the mean (kurtosis = 0—normal distribution). Table 5 presents the detailed results.

Analyzing the number of defibrillations performed with an AED at the scene of the incident, we obtain the following results: the mean is 1.51 (SD = 1.16), the median is 1, min. = 0, max. = 4. Based on the descriptive statistics of the studied variable, most of the results are smaller than the mean (positive skewness), with the indication of the value of the feature less concentrated at the mean (negative kurtosis) (Table 6).

Based on the data collected, on-scene ROSC occurred for only 27 AED interventions (22.50%).

#### 3.3.5. Analysis of Handover of a Patient after OHCA to the Medical Rescue Team

When presenting the rescue operation conducted by the paramedic team, one should start with the analysis of the ambulance arrival time at the scene. From the already collected data, the average time from the moment of arrest of a patient to the moment to EMS arrival was already 7 from 719.72 s (11 min 58 s) with an already 279.98 s (4 min 39 s). The fastest EMS arrived after 320 s (3 min 20 s) and the latest after 1523 s (25 min 23 s). The fastest result was indicative of the closest EMS location to the incident site. The median for the presented variable was 658 s (10 min 58 s). Based on descriptive statistics, most of the results are less than the median (positive skewness) and the number of values is less than 11 min 58 s, a number that indicates many of the values are positive kurtosis.

By subjecting EMS arrival times after receiving a call from an emergency call center to analysis, we get the following results. The fastest EMS arrived after 240 s (4 min) and the latest after 1200 s (20 min). The fastest result was indicative of the closest location of the EMS stationed relative to the scene. The median for the presented variable was 530 s (8 min 50 s). Based on descriptive statistics of the studied variable, most of the results are smaller than the mean (positive skewness), i.e., below 9 min 24 s, indicating the existence of many values close to the mean (positive kurtosis).

When subjected to the analysis of the time of arrival of the Emergency Medical Service relative to the use of the AED, it was noted that the mean time was 469.47 s (7 min 49 s) with SD = 209.13 (3 min 29 min). Relative to the study value, the fastest EMS arrived 131 s (2 min 12 s) after AED use and the latest after 960 s (16 min). The fastest result indicated the closest place of ZRM stationing in relation to the place of event. The median for the presented variable was 479 s (7 min 59 s). Based on descriptive statistics of the studied variable, most of the results were smaller than the mean (positive skewness), i.e., below 9 min 24 s, indicating the value of the feature less concentrated at the mean (negative kurtosis) (Table 7).

Analyzing the activities of the emergency medical services at the scene, it was noted that heart rhythm monitoring was used in each victim. In 48 rescue actions, 1 heart rhythm was recorded in the medical emergency card (40% of cases). In another 32 cards, 2 heart rhythms were entered (26.67%). In 40 cards, no rhythm was marked (33.33%). Based on the 48 cards collected, we can assess which rhythm was predominant: VF—n = 15, 31.25%; VT—n = 2, 4.17%; AS—n = 14, 29.17%; PEA- n = 1; 2.08%; sinus rhythm—n = 16; 33.33%.

The actions taken by the paramedics at the scene were determined by the patient’s condition. While carrying out advanced resuscitation procedures, medical rescue teams performed airway provision, chest compressions, bag-expandable ventilation or implemented ventilator therapy, secured intravenous access, and administered pharmacotherapy. In 10 interventions (8.33%), the patient was pronounced dead at the scene. The remaining number of victims were transported to the appropriate medical facility.

From the study material collected, 16 OHCAs using AEDs (13.33%) resulted in patient survival of more than 30 days.

## 4. Discussion

The aim of the study was to analyze the way and correctness of using public external defibrillators in Poland, taking into account the 10-year period between 2008 and 2018.

The largest number of AEDs is located in the Mazovia Province (area—35,558 km^2^; population—5,403,412; provincial city—Warsaw) and in the Małopolska Province (area—15,183 km^2^; population—3,400,577; provincial city—Kraków) [6]. Comparing the data with those published on the “Rescue with Heart” website [7] the data are similar. According to the coordinators of the project, in 2017 there was the highest number of active AEDs in the Mazowieckie Province. Silesian province recorded a similar rate. The second place was taken by the Małopolska province. When analyzing the provinces with the highest number of stationary AEDs, the order was identical. There are no other data available to compare the presented results with. Taking into account the cities with the highest number of AEDs the order was as follows: Warsaw, Kraków, Katowice, Bydgoszcz, and Łódź. The result is not accidental and incomprehensible, as the presented cities are the seats of province offices. They cover a large area and are inhabited by a significant number of people. Additionally, they have significantly developed industry, education, and administration. Based on the study by Ślęzak [8] and coordinators of the project “Save with Heart” [9] the results are identical. Scientific reports on the location of AEDs in Polish cities were published by Cacko et al. [10] in Warsaw, Żuratyński et al. [11] in Bydgoszcz and Pogorzelczyk et al. [12] in Tricity (Gdańsk, Gdynia, Sopot). Detailed localization data can be found in widely available phone applications or websites i.e., “Save with Heart”,—AED Map (http://www.ratujzsercem.pl/; accessed on 2 October 2020) [13]; AEDMAP (https://www.stayingalive.org/; accessed on 2 October 2020) [14]; AED + You = Life Campaign (https://www.aedplusty.pl/; accessed on 2 October 2020) [15]; AED Project (https://projektaed.pl/; accessed on 2 October 2020) [16]. Due to formal and legal considerations, specific models of AEDs located in public places will not be presented, only their most important technical parameters. A total 63% of the surveyed units answered which company produced the localized AED, and 37% stated only that it was an automatic device. Based on the review of literature and registers of AEDs as well as own observations, semi-automatic devices using biphasic discharge impulse dominate. They may differ only in the waveform, e.g., ascending biphasic SCOPE (optimized biphasic waveform adjusts the energy, slope, and impulse of the discharge to the patient’s impedance); straight biphasic, low energy. The device is certified safe for defibrillation on wet and metal surfaces and resistant to external factors such as drop, water, dust, pressure. This is very important because it can be used in such conditions many times. In terms of size and weight of the device, the dominant devices are up to 2 kg in weight and reach dimensions of 18 cm × 22 cm × 6 cm. The most common AED performs defibrillation in an adult with an energy of 150 J at 32 A, while in children with an energy of 50 J at 19 A. Adult defibrillation: nominal current of 32 amps. This is in accordance with ERC and AHA guidelines. Other technical data are not significant.

The surveyed entities confirmed that AEDs were used 65 times, in 60 locations, during the surveyed period 2008–2018. In 55 cases, the rescue was performed by a unit employee (84.62%), while only 10 were performed by a bystander (15.38%). Only 23 units described the circumstances of AED use.

After each use of the AED, the device should be serviced, the battery and adhesive electrodes replaced. For 34 locations where an AED was used, the unit reported the fact to the distributor/manufacturer of the device (56.67%). Every unit that reported AED use received any assistance from the AED distributor/manufacturer. The assistance consisted of replacing the leads, servicing the device, and reading out the record of the resuscitation action. Contrasting these data with those obtained from manufacturers and distributors of AEDs in Poland, AED managers are not obliged to report AED use. According to the distributors, buying new electrodes or replacing batteries does not indicate that the AED has been used, but may be a result of the elements becoming obsolete. Reiterating the most common reasons for not reporting use, distributors noted that entities do not want to publicize incidents as they may have a bad reputation for accidents at work.

Sudden cardiac arrest is more common among men. This is supported by many studies. The male gender is at risk for all heart diseases [1,3,17,18,19,20]. In the material presented here, there is no deviation from global trends. A total 87.5% of AED interventions involved males. When analyzing the age group, it was observed that the highest rate of AED use occurred in the age group of 50–60 years (31%), which is included in the mean (57.27 years) and median (56.5 years). Again, this result can be correlated with the risk group and epidemiological data of SCA [1,3,17,18,19,20].

The largest group who experienced OHCA with AED intervention were travelers by various modes of transportation (30%). This is a result of high availability of defibrillators in the buildings of air, railroad, bus, or other public transport infrastructure. This group includes cases of AED use on board trains, in airports, and at railroad stations. According to the guidelines of ERC or AHA these are the places with the highest risk of OHCA, so the aim should be to have min. Therefore, there should be at least 1 automated defibrillator. This was confirmed by Caffrey et al. [21], O’Rourke et al. [22], Page et al. [23] and Weaver et al. [24], who published studies on AED use at airports and on board aircraft. Another group consisted of customers of retail, banking, or office establishments (23.33%) and employees (22.5%). These data can be compared with the profile of the place of defibrillator use. Most cases were on public transport infrastructure (n = 41; 34.17%), followed by places of work ( n =21; 17.5%) and places of public service (n = 20; 16.67%). Again, this is supported by the ERC and AHA guidelines as high-risk places.

The first person on the scene is of major importance in reducing OHCA mortality. The implementation of resuscitation depends on this person. Apart from notifying the emergency services and initiating resuscitation, it is important to provide and correctly use the AED. In the study group, it was most often an employee (80%) of one of the places. Security personnel were predominant. Besides them there were policemen, medical staff, hotel staff, streetcar drivers, and swimming pool attendants. Most of them were people who were responsible for AED in a given moment, had training in its use and were obliged to use it in a given moment. In one of the cases of AED use in Kraków, which took place in a library, the defibrillator was used thanks to the efficiency and self-control of the security guard. Only 20% were casual witnesses. Most often they were paramedics, medical students, and qualified first aid workers. A thorough comparison of the results with the literature is lacking, as only “first responders” or “witnesses” appear (witness), without a detailed characterization of that person.

When presenting the actions of a witness to an event with an OHCA victim, it was noted that in 63.33% the mechanism of cardiac arrest was defibrillatory (VT or VF), 30.83% nondefibrillatory or sinus rhythm was recognized. In 5.83%, no data were obtained. Based on scientific data, the most common SCA rhythm is ventricular fibrillation or ventricular tachycardia without a pulse. Each time the European Resuscitation Council or American Heart Association recommends the fastest possible defibrillation, which performed within 3–5 min from cardiac arrest determines the survival rate of 50–70%. It is worth noting that with every minute of delay in implementation of AED, the probability decreases by 10–12% [25,26,27,28,29]. As mentioned, the rapid response of the witness to the event is important. On the basis of 33 documented resuscitation actions with the use of AED it can be stated that the average time that elapsed from SCA to the use of AED was 3 min 22 s, which was consistent with the ERC and AHA guidelines. The longest time elapsed from SCA to AED use was 10 min. In this case, the victim was defibrillated 2 times at the scene, then transferred to the emergency medical team, but did not receive ROSC. What reasons might have contributed to some of the delays in providing the AED? These include the witness’s lack of knowledge of the location of the AED, improper signage of the location, or the medical dispatcher’s lack of knowledge of the location. As stated in the 2015 ERC resuscitation guidelines, the medical dispatcher should have knowledge of the current distribution of AEDs in their area. Telec et al. [27] investigated the assessment of actual AED availability and the assessment of possible sources of defibrillation delays in Poznań, Łódź, and Warsaw. From a selected group of 200 sites, they chose 78 sites and sent volunteers who had no knowledge of the location of AEDs in that site. Based on the OHCA simulation, the volunteers had to respond appropriately including, but not limited to, using the AED. The devices were located within a range of 2–163 m from the scene. The average total device delivery time was 96 s (1 min 36 s) with a maximum of 144 s (2 min 24 s). Delays that may occur in obtaining an AED were also assessed. These included only discussion with the person responsible for the AED (safety officer, staff, etc.) (mean time—16 s, max 49 s). The authors drew their conclusions, i.e., the recommended time for early defibrillation was below 3 min and there were some reasons that could affect the delay of defibrillation, e.g., badly marked places, lack of unrestricted access to AEDs (kept under the care of personnel or in cabinets).

Summing up the resuscitation actions performed, the average total time of AED operation on the scene was 7 min 34 s (+/−3 min 10 s). The minimum time of AED operation was 1 min 11 s, the maximum—15 min 23 s. Analyzing the number of defibrillations performed with AED at the scene, the average was 1.51 and the maximum was 4 defibrillations. Non-defibrillation rhythms were recognized in the total of 8 events (AS – n = 5, 15.15%; PEA – n = 3; 9.09%). The time elapsed from the end of rhythm analysis and defibrillation recommendation to the triggering of the electrical impulse was on average 7 s (SD=1 s), which is confirmed by the device specifications. The time from the end of CPR to readiness for discharge was 8 s [30].

The goal of resuscitation procedures is to achieve ROSC. As is well known, rapid defibrillation is important in addition to chest compressions and ventilation. On the basis of such a small group, it cannot be concluded whether the return of spontaneous blood circulation is related to AED and public access defibrillation (PAD). In the study group, ROSC at the scene occurred in only 27 cases, with missing data from 58 cases. This is a topic that requires further research. Citing reports by Dwyer and Dennett [31], the authors concluded that the use of AEDs in patients after cardiac arrest was not associated with improved survival. This study cannot be compared to the results of this paper because Dwyer and Dennett’s inference was for in-hospital cardiac arrest and in-hospital AED use.

Analyzing the time of arrival relative to the call and disposition received from the emergency notification centers, it was noted that the mean time was 564.66 s (9 min 24 s) with SD = 279.98 (4 min 39 s). The fastest EMS arrived after 240 s (4 min) and the latest after 1200 s (20 min). The fastest result indicated the nearest EMS station to the incident site. When analyzing the time of arrival of the first responders relative to the use of the AED, it was noted that the mean time was 469.47 s (7 min 49 s) with SD = 209.13 (3 min 29 s). Relative to the study value, the fastest EMS arrived 131 s (2 min 12 s) after AED use and the latest after 960 s (16 min). The fastest result indicated the closest location of EMS in relation to the incident site. The time of arrival of the first responders to the scene of the accident was within the standards established by the National Medical Rescue Act [32]. Recalling the study by Zijlstra et al. [33], the witness to the event used the AED on average 2 min 39 s before the arrival of the emergency medical service and for 10.5% performed the discharge less than 6 min after the call. Hallstrom et al. [34] noted that the average time from the 911 call to the first rhythm analysis was almost 3 min shorter.

From the collected evidence, 16 OHCAs with AEDs (14.55%) had a patient survival of more than 30 days. A review of the literature showed a median overall survival of 40% in OHCA patients treated with PAD [35].

## 5. Conclusions

The predominant location of AED use is in public transportation facilities, and the injured party is the traveler. AED use in non-hospital settings is more common in male victims aged 50–60 years. Owners of AEDs inadequately provide information about their use. The documentation that forms the basis of the emergency medical services intervention needs to be refined. There is no mention of resuscitation performed by a witness of an event or of the use of an AED. In addition, Poland lacks the legal basis for maintaining a register of automated external defibrillators. There is a need to develop appropriate documents to determine the process of reporting by the owners of the use of AEDs in out-of-hospital conditions (OHCA).

## Figures and Tables

**Table 1:** Basic descriptive statistics on the age of injured people.

	Mean	Median	Minimum	Maximum	Standard Deviation	Skewness	Kurtosis
AGE	57.27	56.50	18.00	93.00	17.04	0.10	−0.41

**Table 2:** Profile of the person on whom the AED has been used (n = 120).

Victim Profile	n	%
traveler by bus, tram, rail, air transport	36	30.00
pedestrian	10	8.33
employee	27	22.50
client/supplicant	28	23.33
tourist	5	4.17
ward of a social welfare home	9	7.50
sport competitor	1	0.83
prisoner	4	3.33

**Table 3:** Profile of the place where the AED has been used (n = 120).

Place Profile	n	%
Public space, i.e., pavement, market, car park	8	6.67
Place of work (factory, warehouse, mine, steelworks)	21	17.50
Shopping centre (estate shop, multi-store)	9	7.50
Public transport infrastructure (train/bus station, railway station/bus stop, metro stations)	29	24.17
Sports centre (swimming pool, playground, stadium, sports hall, gym)	5	4.17
Airport/seaport	10	8.33
Bank	2	1.67
Cultural and entertainment institution (cinema, theatre, opera, museum, art gallery, library)	2	1.67
Place of temporary accommodation (hotel, holiday home, hostel, hostel)	3	2.50
Authority /Institution	5	4.17
Urban/interurban transport (tram, bus, trolleybus, train, metro) -mobile	2	1.67
Place of worship (church, monastery, place of religious assembly)	2	1.67
State, governmental, territorial administration (ministry, town/municipality/county office)	4	3.33
Educational entities (school, university)	1	0.83
Social welfare home	9	7.50
Prison	4	3.33
Lakeside beach	4	3.33

**Table 4:** Basic descriptive statistics on the number of defibrillations and the number of rhythm analysis (n = 33).

	Mean	Median	Minimum	Maximum	Standard Deviation	Skewness	Kurtosis
Time from SCA to AED use [s]	222.73	180.00	60.00	600.00	121.98	1.69	3.01
Time from switching on the AED to electrode sticking [s]	35.36	22.0	4.00	115.00	31.17	1.05	0.34
Number of AED rhythm analyses	2.79	2.00	1.00	6.00	1.17	0.94	0.60

**Table 5:** Basic descriptive statistics on AED operation time at scene of the incident (n = 33).

	Mean	Median	Minimum	Maximum	Standard Deviation	Skewness	Kurtosis
AED operation time [s]	454.12	478.00	131.00	923.00	190.62	0.39	0.00

**Table 6:** Basic descriptive statistics on the number of defibrillations performed with using an AED (n = 33).

	Mean	Median	Minimum	Maximum	Standard Deviation	Skewness	Kurtosis
number of AED defibrillations	1.51	1.00	0.00	4.00	1.16	0.34	−0.78

**Table 7:** Basic descriptive statistics on the number of the quantitative variables studied regarding EMS interventions.

	Mean	Median	Minimum	Maximum	Standard Deviation	Skewness	Kurtosis
Time from SCA to EMS arrival [s]	719.72	658.00	320.00	1523.00	279.98	0.92	0.62
Time from call to EMS arrival [s]	564.66	530.00	240.00	1200.00	235.60	0.85	0.24
Time from AED use to EMS arrival [s]	469.47	479.00	131.00	960.00	209.13	0.48	−0.16

## Data Availability

Data sharing not applicable.
